# Annular lesions in an HIV-positive male

**DOI:** 10.1016/j.jdcr.2024.11.043

**Published:** 2025-01-28

**Authors:** Nicole Natarelli, Elisha Myers, Wei-Shen Chen, Paul Rodriguez-Waitkus

**Affiliations:** a University of South Florida, Morsani College of Medicine, Tampa, Florida; b Charles E. Schmidt College of Medicine at Florida Atlantic University, Boca Raton, Florida; c Department of Dermatology and Cutaneous Surgery, University of South Florida, Tampa, Florida

**Keywords:** annular, histopathology, HIV, immunostain, syphilis

## History

**Fig 1:**
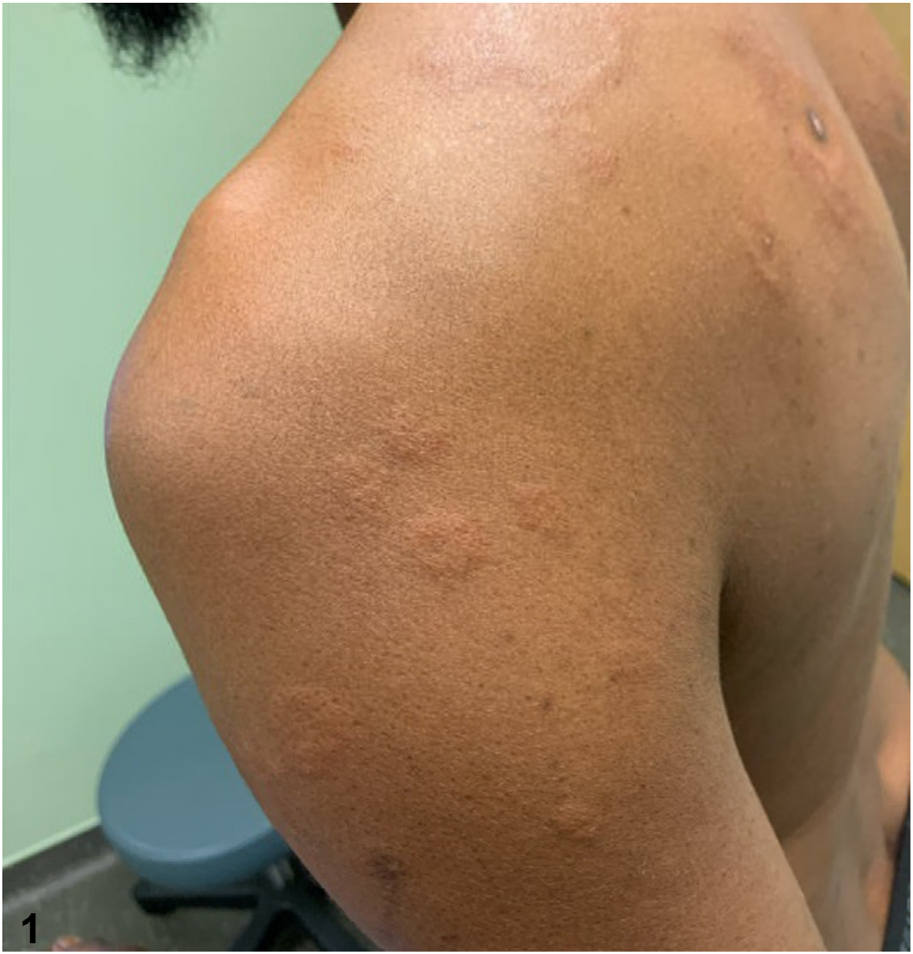

**Fig 2:**
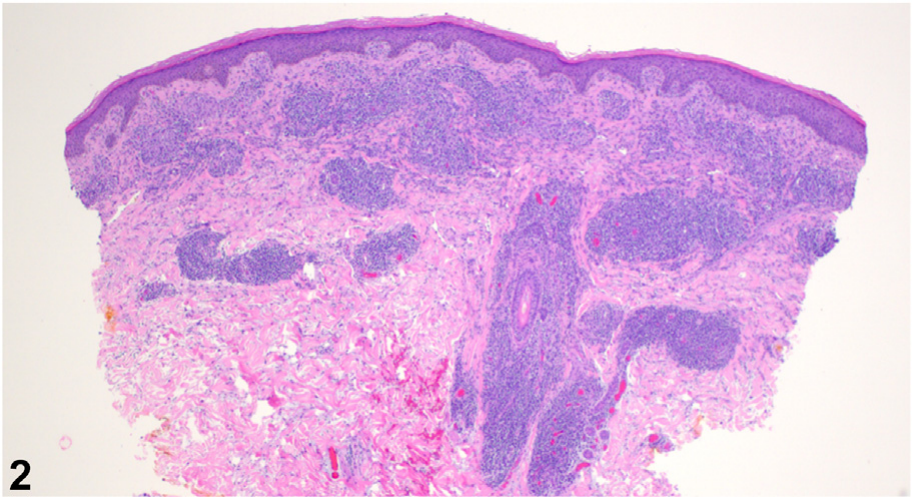

**Fig 3:**
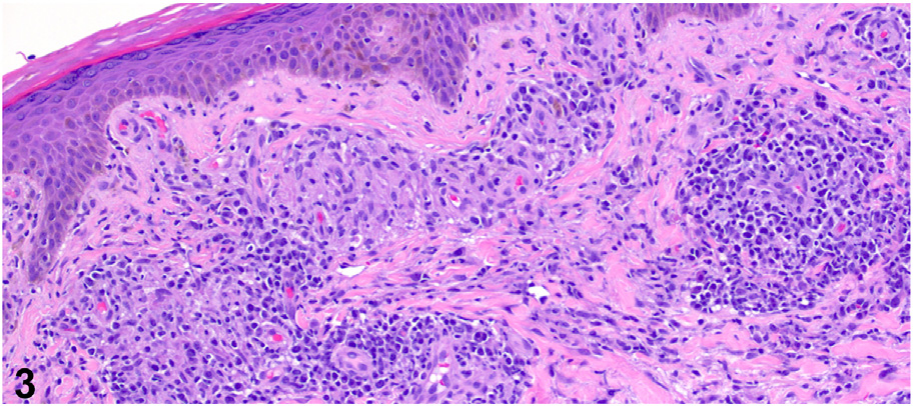

**Fig 4:**
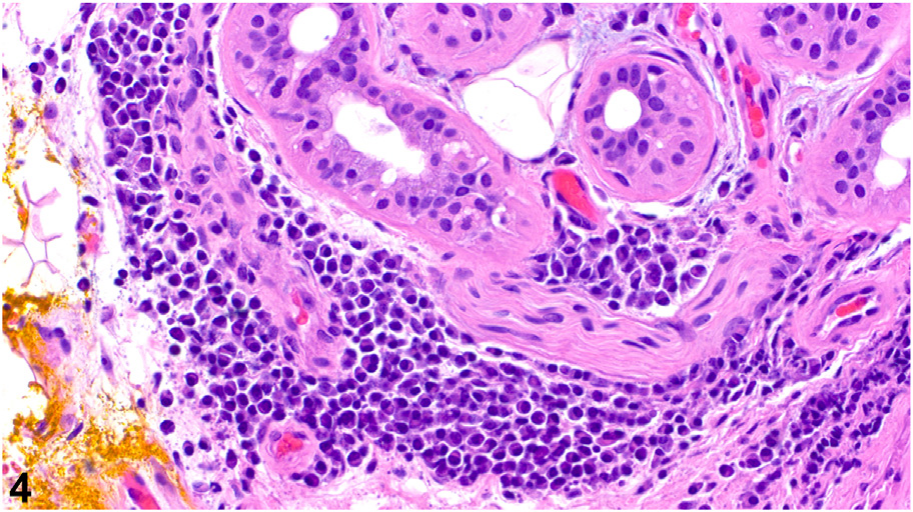

A 54-year-old male with a past medical history of human immunodeficiency virus (HIV) well-controlled with antiretroviral therapy presented with annular plaques on the trunk and extremities. The eruption began rapidly about 1 month prior with associated pruritus and a stinging sensation at onset. He denied new medications, recent illnesses, blisters, or bleeding. A total body skin examination revealed multiple annular, edematous pink papules and plaques, some with central crust, on the bilateral upper extremities, posterior trunk, and right anterior ankle, sparing the palms and soles (Fig 1). No vesicles or bullae were observed. Biopsies of the right anterolateral shoulder and the left anterior forearm revealed superficial/deep perivascular and periadnexal lymphoplasmacytic infiltrate (Fig 2), well-formed granulomas (Fig 3), and numerous plasma cells (Fig 4).

**Question 1: What is the most likely diagnosis?**

A. Granuloma annulare
B. Cutaneous sarcoidosis
C. Secondary syphilis
D. Annular urticaria
E. Cutaneous plasmacytoma

**Answers:**

**A.** Granuloma annulare – Incorrect. Granuloma annulare shows a dermal palisading histiocytic infiltrate surrounding areas of collagenolysis with increased mucin deposition.
**B.** Cutaneous sarcoidosis – Incorrect. Sarcoidosis shows discrete collections of epithelioid histiocytes with sparse lymphocytic infiltrate (so-called “naked” granulomas).
**C.** Secondary syphilis – Correct. Secondary syphilis is the great inflammatory mimicker. It can manifest as different inflammatory patterns including psoriasiform, lichenoid, granulomatous, and superficial/deep perivascular dermatitides with an increased plasmacytic infiltrate. Some patients can present with localized or diffuse alopecia, clinically and histologically resembling alopecia areata. Clinically, our patient presented with pruritic annular plaques. A 10-year retrospective study (*n* = 307) found 25.8% of patients depicted atypical cutaneous presentations, most commonly in the secondary stage.^1^ Annular, pruritic, and scaly plaques with a psoriasiform appearance were the most common atypical presentations of secondary syphilis.
**D.** Annular urticaria – Incorrect. While annular urticaria may be considered due to the itching and stinging sensation upon onset and the edematous morphology of the lesions, annular urticaria usually presents with transient edematous plaques that resolve within 24-48 hours. In addition, central crusting is not typical of urticaria, and histopathology findings more typically show superficial dermal edema, rather than plasma cells and granulomatous features.
**E.** Cutaneous plasmacytoma – Incorrect. Clinically, cutaneous plasmacytoma more often presents as firm, red, or violaceous nodules or plaques. Many patients with cutaneous plasmacytoma also experience bone pain.

**Question 2: The differential diagnosis includes (1) granuloma annulare, (2) sarcoidosis, (3) annular urticaria, (4) secondary syphilis, and (5) cutaneous plasmacytoma. Which of the following immunostains or special stains would be the least helpful in supporting or ruling out a diagnosis included in this differential?**

A. CD68
B. CD4
C. Treponemal immunostain
D. Prussian blue
E. CD138

**Answers:**

**A.** CD68 – Incorrect. While not routinely utilized in the diagnosis of granulomatous conditions, CD68 stain for macrophages and multinucleated giant cells can highlight the presence of these cells in a granulomatous condition and may support the final diagnosis. In summary, a CD68 stain may be helpful to support or rule out a granulomatous condition, such as cutaneous sarcoidosis.
**B.** CD4 – Incorrect. In some cases of secondary syphilis, the density of inflammatory cells can be concerning for a cutaneous lymphoma/lymphoproliferative disorder. CD4 stains are often used to assess the ratio of CD4+ to CD8+ T cells in inflammatory conditions. An elevated/abnormal CD4/CD8 T-cell ratio indicates an atypical lymphocytic infiltrate and may suggest a lymphoproliferative disorder. In summary, a CD4 stain may be helpful to support or rule out a lymphoproliferative disorder.
**C.** Treponemal immunostain – Incorrect. The treponemal immunostain can detect the presence of *Treponema pallidum* spirochetes, suggesting secondary syphilis. In this case, treponemal immunostain highlighted numerous intraepidermal and superficial perivascular organisms to support the diagnosis. Secondary syphilis is the great inflammatory mimicker. It can manifest as different inflammatory patterns including psoriasiform, lichenoid, granulomatous, and superficial/deep perivascular dermatitides with an increased plasmacytic infiltrate. Some patients can present with localized or diffuse alopecia, clinically and histologically resembling alopecia areata.
**D.** Prussian blue – Correct. The Prussian blue stain, which is used to detect hemosiderin deposits, would not be useful in this case. The clinical presentation and histopathological findings do not suggest a condition involving iron metabolism or increased iron deposition. In summary, the Prussian blue stain is the least helpful stain listed due to the lack of iron-mediated pathology on the differential.
**E.** CD138 – Incorrect. CD138 staining is an immunohistochemical test for plasma cells and is used to evaluate abnormal numbers and distribution of these cells in biopsies. As cutaneous plasmacytoma is on the differential diagnosis, a CD138 stain may be helpful to support or rule out this diagnosis.

**Question 3: A treponemal immunostain highlights intraepidermal and perivascular spirochetes. What is the next best step in management?**

A. Administer 1 dose of intramuscular penicillin
B. Prescribe oral corticosteroids for the skin lesions
C. Initiate antiviral therapy for HIV
D. Order a chest X-ray to check for pulmonary involvement
E. Start oral doxycycline for 14 days

**Answers:**

**A.** Administer 1 dose of intramuscular penicillin – Correct. Intramuscular penicillin is the first-line treatment for secondary syphilis as it directly targets and eradicates the *Treponema pallidum* bacteria.^2^ While 3 doses were previously recommended for HIV and secondary syphilis coinfection, no therapeutic benefit has been demonstrated. Thus, patients should receive 1 dose of intramuscular penicillin regardless of HIV status.
**B.** Prescribe oral corticosteroids for the skin lesions – Incorrect. Corticosteroids reduce inflammation and may mitigate pruritus, but they do not address the underlying cause of the infection, the spirochete bacterium *Treponema pallidum.*
**C.** Initiate antiviral therapy for HIV – Incorrect. This patient is already receiving antiretroviral therapy, and his CD4 counts are within normal limits.
**D.** Order a chest X-ray to check for pulmonary involvement – Incorrect. While a chest X-ray may be required in the workup of sarcoidosis, it is not routinely utilized in the management of syphilis. The next best step in management should include the treatment of secondary syphilis.
**E.** Start oral doxycycline for 14 days – Incorrect. Oral doxycycline is an alternative treatment for syphilis, but it is reserved for penicillin-allergic patients.^2^ Rather, the preferred treatment for secondary syphilis is intramuscular penicillin.

## Conflicts of interest

None disclosed.
